# Drug-resistant genes, virulence characteristics, and molecular typing of clindamycin-resistant *Streptococcus agalactiae* in late pregnancy

**DOI:** 10.3389/fcimb.2025.1721174

**Published:** 2026-01-20

**Authors:** Meng Yu, Ting Yu, Shui Yu, Li Li, Shuang Chen, Yujie Wang, Kun Wang

**Affiliations:** 1 Clinical Laboratory, Qingdao Municipal Hospital, Qingdao, China; 2 Clinical Laboratory, Qingdao Women and Children’s Hospital, Qingdao, China; 3 Clinical Laboratory, Jinan Maternity and Child Care Hospital Affiliated to Shandong First Medical University, Jinan, China

**Keywords:** drug resistance, molecular typing, pregnant women, *Streptococcus agalactiae*, virulence genes

## Abstract

**Background:**

*Streptococcus agalactiae* increases the risk of adverse pregnancy outcomes and neonatal infections. Clindamycin is a key alternative for intrapartum prophylaxis in penicillin-allergic women, but the prevalence of clindamycin-resistant *S. agalactiae* is increasing, posing a significant clinical challenge.

**Methods:**

A total of 178 strains isolated from tertiary hospitals in Jinan and Qingdao, Shandong Province, China, were characterized using antimicrobial susceptibility testing, whole-genome sequencing, multilocus sequence typing, serotyping, and analysis of resistance and virulence genes.

**Results:**

All strains were susceptible to penicillin, ampicillin, linezolid, vancomycin, and tigecycline. In contrast, resistance rates to erythromycin, levofloxacin, and tetracycline were 95.5%, 60.1%, and 56.7%, respectively. Six serotypes and 15 sequence types belonging to eight clonal complexes were identified. Notable regional differences were observed. The Ib-ST10-CC12 lineage dominated in Jinan, whereas V-ST529-CC327 was predominant in Qingdao. The resistance gene *mreA* was ubiquitous (100%), followed by *ermB* (80.3%). The key virulence genes *cylE*, *hylB*, and *pavA*, were detected in all strains. *fbsA* (99.4%), the alpha protein family (98.9%), *cfb* (98.3%), the Pilus Island gene cluster (94.9%), and *lmb* (92.7%) were also highly prevalent. The two major clindamycin resistance genes, *erm* and *lnuB*, exhibited distinctly different enrichment patterns among *S. agalactiae* clonal complexes, despite a certain overlap in CC19 and CC327. Specifically, *erm* was significantly enriched in CC12 (serotype Ib), CC19 (III/V), and CC327 (III/V). In contrast, *lnuB* was predominantly restricted to CC19 and CC327, where it defined a unique phylogenetic subcluster. Significant differences in resistance and virulence gene profiles were observed across different clonal complexes.

**Conclusion:**

Clindamycin-resistant *S. agalactiae* in late-pregnancy women in Shandong Province, China exhibits a broad resistance spectrum, diverse molecular types, and significant regional heterogeneity. These findings underscore the need for continued surveillance and region-specific strategies for preventing neonatal *S. agalactiae* infections.

## 1 Introduction

*S. agalactiae* is a significant cause of perinatal infections and poses enormous challenges to global public health owing to its epidemiological characteristics and the evolution of drug resistance. Epidemiological data indicate that *S. agalactiae* colonization rates in the genital tract of women in late pregnancy reach 10%–30% (Russell et al., 2017).It is the primary pathogen that causes early onset neonatal invasive disease, including sepsis and meningitis, with an incidence ranging from 0.55 to 2.59 cases per 1000 live births (Absalon et al., 2021). *S. agalactiae* infection is closely associated with adverse pregnancy outcomes, such as chorioamnionitis and preterm birth (Gaddy et al., 2024). Currently, the US Centers for Disease Control and Prevention (CDC) and international guidelines recommend penicillin-based antibiotics for pregnant women with *S. agalactiae* colonization during labor to prevent infection; clindamycin or erythromycin is commonly used as an alternative treatment (Cagno et al., 2012). However, widespread antibiotic use has led to increasing *S. agalactiae* resistance, particularly to erythromycin and clindamycin, which poses significant challenges for clinical prevention and treatment.

The pathogenicity of *S. agalactiae* is associated with its molecular characteristics. Globally, serotypes la, lb, III, and V are the predominant circulating types (Nanduri et al., 2019), with serotype III strains associated with over 60% of neonatal meningitis cases (Wang et al., 2023). Molecular mechanism studies have indicated that these highly pathogenic strains typically harbor adhesion- and invasion-related genes, such as *lmb* and *scpB* (Li et al., 2025), along with *HvgA*, a signature virulence factor of the highly virulent clonal complex 17 (CC17) (Aznar et al., 2024). Virulence genes exhibit distinct clonal specificities. CC17 accumulates *HvgA* and the Pl-2b virulence island (Almeida et al., 2017), whereas CC19 tends to carry the Pl-1 virulence island (Nie et al., 2018). Furthermore, virulence factors, such as the fibrinogen-binding protein Srr1/2 and hyaluronidase hylB, participate in bacterial colonization and invasion through distinct mechanisms (Ma et al., 2025). In contrast, variations in capsular polysaccharide synthesis genes directly affect immune evasion and vaccine efficacy (Richardson et al., 2025).

*S. agalactiae* exhibits complex phenotypic characteristics and molecular diversity in its resistance mechanism. Clindamycin resistance primarily manifests in three patterns: constitutive macrolide-lincosamide-streptogramin B(cMLSB), inducible macrolide-lincosamide-streptogramin B (iMLSB), and the uncommon CRES phenotype (also referred to as L-type, defined as clindamycin-resistant and erythromycin-susceptible). The cMLSB phenotype is mediated by the *ermB* gene, conferring intrinsic resistance through 23S rRNA methylation. The iMLSB phenotype requires the *erm* gene expression, which is induced by erythromycin. The CRES phenotype is mediated by the *lnu(B)* or *lsa(C/E)* (He et al., 2022). A Polish study indicated that among 421 clinical isolates, the distribution proportions of these three phenotypes were 78.2%, 14.9%, and 6.9%, respectively, with *ermB* being significantly associated with serotype V (odds ratio = 4.3) (Kamińska et al., 2024). Tetracycline resistance primarily involves *tetM* (ribosomal protection) and *tetO/tetS* (efflux pump) mechanisms. Quinolone resistance mainly stems from mutations in *gyrA* and *parC*, particularly the dual mutation pattern of gyrA_S81L combined with parC_S79Y (El-Sagheir et al., 2025). *S. agalactiae* drug resistance is significantly associated with specific clonal complexes (CCs) and serotypes. A pioneering analysis of South American *S. agalactiae* populations based on whole-genome sequencing revealed that CC19 isolates (predominantly type III) harbored the highest density of virulence and resistance determinants (Kovacec et al., 2025).

The colocalization of resistance and virulence genes on mobile genetic elements forms unique “virulence-resistance” gene clusters (Shang et al., 2025). This genetic combination enhances the pathogenicity of the strain and facilitates the transmission of resistance, posing significant challenges to clinical management.

However, previous studies have indicated significant geographical variations in *S. agalactiae* drug resistance patterns, serotype distribution, and virulence gene combinations. Systematic molecular epidemiological research on *S. agalactiae* in late pregnancy remains scarce, particularly in the Asia-Pacific region. As late pregnancy *S. agalactiae* colonization is a primary risk factor for neonatal early onset *S. agalactiae*, comprehensive research on antibiotic resistance, virulence characteristics, and molecular typing in this specific population is significant. The purpose of this study is to conduct targeted investigations to elucidate the key characteristics of *S. agalactiae* colonization in pregnant women during late pregnancy within specific regions. Through systematic detection and analysis of drug-resistance phenotypes, resistance genes, serotypes, molecular typing (including sequence types and CCs), and virulence genes, this study aims to define the epidemiological patterns and molecular characteristics of *S. agalactiae* in this population. The findings are expected to address the existing gap in molecular epidemiological data on *S. agalactiae* during late pregnancy in the Asia-Pacific region and to provide an evidence-based foundation for developing region-specific screening strategies, optimizing prevention and control measures for neonatal early-onset *S. agalactiae* infections, and informing the clinical selection of antimicrobial agents.

## 2 Materials and methods

### 2.1 Ethics statement

Informed consent was obtained from all participants for strain collection, and the study was approved by the Ethics Committees of Jinan Maternity and Child Care Hospital Affiliated to Shandong First Medical University and Qingdao Municipal Hospital.

### 2.2 Bacterial strain collection and identification

*S. agalactiae* strains were collected from prenatal screening of pregnant women in the third trimester (35–37 weeks of gestation) at Grade A tertiary hospitals in Jinan and Qingdao, Shandong Province, between March and October 2024. Without using a vaginal speculum, a sterile disposable nylon swab was used to collect secretions from the lower one-third of the vaginal canal. The same swab was then used to collect rectal secretions from a site 2–3 cm above the anal sphincter. The swab was subsequently placed into a sterile sealed tube and transported immediately for testing. Following initial culture on 5% sheep blood agar, species identification was conducted using a Bruker MALDI-TOF MS system (Bruker Daltonik GmbH, Germany) in accordance with standard operating procedures. All strains were primary isolates (duplicate isolates from the same patient were excluded), preserved in tryptic soy broth with 20% glycerol, and stored at –80°C. Standardized procedures were maintained throughout to ensure data reliability.

### 2.3 Antimicrobial susceptibility testing

The susceptibility of *S. agalactiae* strains to nine antibiotics (penicillin, ampicillin, linezolid, vancomycin, tigecycline, clindamycin, erythromycin, levofloxacin, tetracycline) was determined using the VITEK 2 system (bioMérieux, Marcy-l’Étoile, France) according to the manufacturer’s instructions. *Streptococcus pneumoniae* ATCC 49619 was used as the quality control strain in each batch to verify accuracy. Results were interpreted according to the Clinical and Laboratory Standards Institute (CLSI) M100-S33 guidelines. The D-test was performed to differentiate constitutive (cMLSB) and inducible (iMLSB) resistance phenotypes. Oxoid antimicrobial disks (Thermo Fisher Scientific, Waltham, MA, USA) containing erythromycin (15 μg) and clindamycin (2 μg) were placed 15–20 mm apart on Mueller-Hinton agar supplemented with 5% sheep blood. After incubation at 37°C with 5% CO_2_ for 18–24 hours, a “D-shaped” inhibition zone around the clindamycin disk indicated the iMLSB phenotype, whereas the absence of inhibition around the clindamycin indicated the cMLSB phenotype.

### 2.4 Whole genome sequencing

Frozen stocks of *S. agalactiae* preserved in 20% glycerol TSB were retrieved from -80°C. A 200 μL aliquot was inoculated into 5 mL of Todd-Hewitt Broth and cultured with shaking at 37°C under 5% CO_2_ for 16–18 hours until the logarithmic growth phase (OD_600_ = 0.6–0.8). A 100 µL aliquot of the revived culture was spread onto 5% sheep blood agar, and after 24 h of incubation under the same conditions, purity was confirmed by colony morphology. For DNA extraction, 1.5 mL of log-phase culture was centrifuged at 12,000 × g for 5 minutes, and the pellet was washed twice with PBS (pH 7.2). Genomic DNA was extracted using the Wizard Genomic DNA Purification Kit (Promega, Madison, WI, USA) and assessed with a NanoDrop 2000 spectrophotometer (Thermo Fisher Scientific) and agarose gel electrophoresis. Qualified DNA samples were sent to BGI Genomics (Shenzhen) for library preparation. Whole-genome sequencing was performed on the DNBSEQ platform. Raw sequencing data were processed with Trimmomatic v0.39 to remove adapters and low-quality reads, and assembled *de novo* using SPAdes v3.9.0 to generate the final genome assembly.

### 2.5 Multilocus sequence typing, CC analysis, serotype identification, and minimum spanning tree construction

MLST was performed using the mlst software (v2.23.0; https://github.com/tseemann/mlst), targeting seven housekeeping genes (*adhP, pheS, atr, glnA, sdhA, glcK, and tkt*) according to the PubMLST typing scheme (https://pubmlst.org/sagalactiae/). The software aligned assembled genome sequences to the PubMLST allele database to assign allele numbers, and sequence types (STs) were determined from allele profiles. CCs were assigned with the goeBURST algorithm implemented in eBURST (v3.0; available at http://eburst.mlst.net). Strains sharing six of seven alleles (6/7 exact match) were grouped into the same CC, while unassigned strains were classified as “non-typeable” (NA). Serotype identification was based on sequence homology of capsular polysaccharide synthesis (cps) genes. Assembled genomes were aligned to a reference database of *S. agalactiae* cps sequences using BLAST+ (v2.15.0; https://blast.ncbi.nlm.nih.gov). Serotypes were assigned when alignments met the following thresholds: e-value ≤ 1e–5, identity ≥ 95%, and coverage ≥ 90%. To visualize genetic relationships among *S. agalactiae* strains, a MST was constructed from core genome single-nucleotide polymorphism (SNPs). SNP calling was performed with Snippy (v4.6.0), excluding SNPs in repetitive regions and mobile genetic elements. The MST was generated with GrapeTree (v2.1; https://github.com/achtman-lab/GrapeTree) using the MSTreeV2 algorithm, where each node represented a strain and inter-node distances corresponded to the number of core genome SNPs.

### 2.6 Data analysis

Antimicrobial resistance gene detection: Resistance genes were identified with the srst2 tool against the CARD database (v4.0.0, website: https://card.mcmaster.ca), using thresholds of ≥ 90% sequence identity and ≥ 90% coverage.

Virulence factor analysis: Virulence factors were identified using the VFDB database (2025 edition, website: http://www.mgc.ac.cn/VFs) with the same srst2 thresholds (≥90% identity, ≥ 90% coverage).

Visualization: A Sankey diagram was generated using the networkD3::sankeyNetwork() function to illustrate relationships among sampling locations (Jinan and Qingdao), serotypes, STs, and CCs. Nodes and links were constructed to reflect strain distributions across these hierarchical categories.

Heatmap analysis: Heatmaps were generated to visualize associations between strains and features. Standardized data were arranged in a numerical matrix, with rows representing strains and columns representing features. The heatmap was generated using the pheatmap package (version 1.0.12) in R, with color gradients showing value distributions. Hierarchical clustering of rows and columns was performed simultaneously using the default Euclidean distance and complete linkage to reveal similarity patterns.

## 3 Results

### 3.1 Bacterial isolates and study population

In total, 178 clindamycin-resistant *S. agalactiae* strains were collected during routine prenatal screening of pregnant women in their third trimester. The ages of the pregnant women ranged from 22 to 46 years, with a median age of 34. Among them, 85 strains were obtained from Jinan City; the age range of the women was 22–40 years, with a median age of 31. Additionally, 93 strains were obtained from Qingdao City; the age range of the women was 22–46 years, with a median of 34.

### 3.2 Antimicrobial susceptibility profiles

Among the 178 strains, the main phenotype was cMLSb, which accounted for 64.0% (114/178). The second was iMLSb, which accounted for 32.6% (58/178) of cases. The proportion of the CRES phenotype was the lowest at approximately 3.4% (6/178).

**Figure 1:**
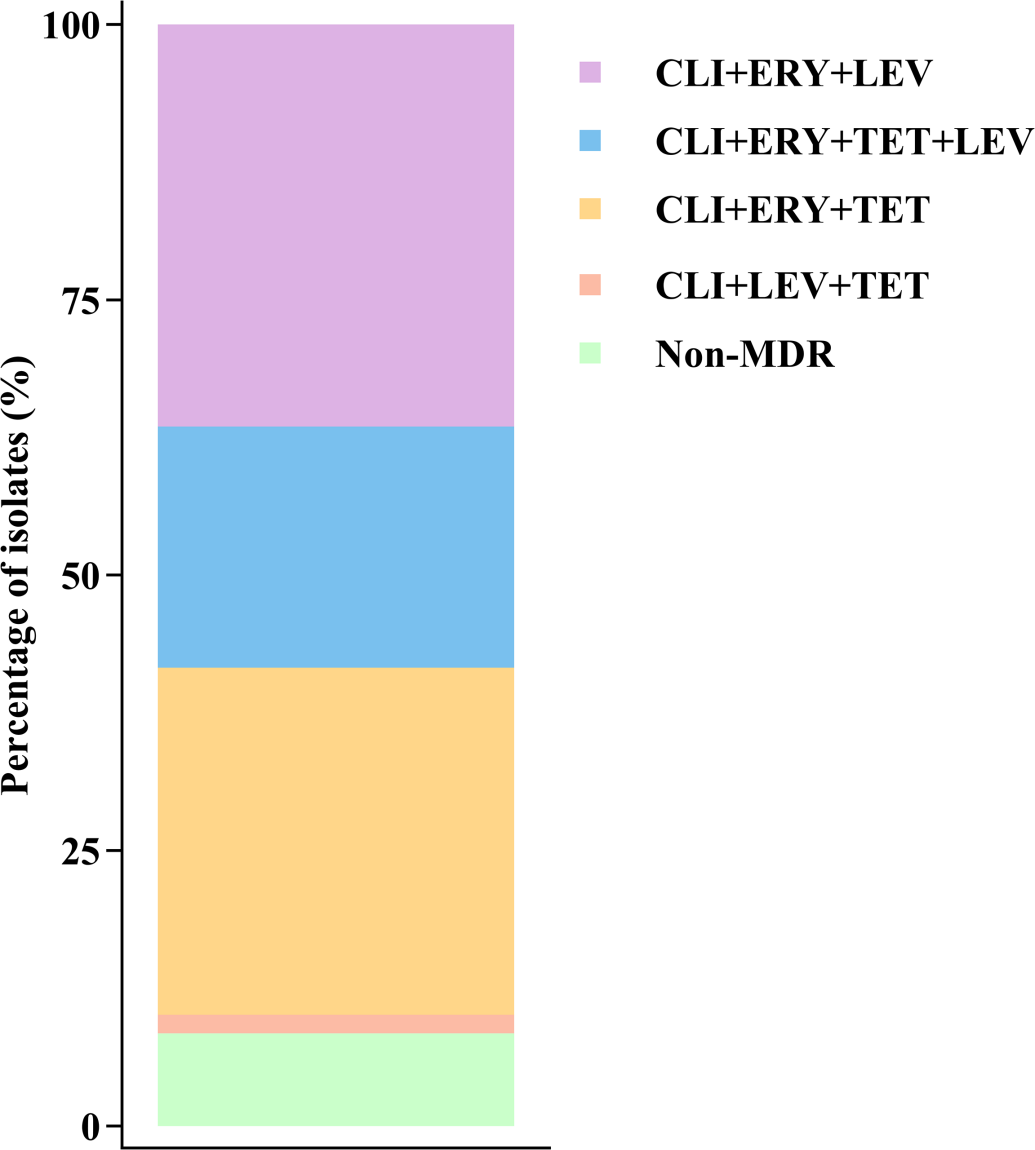
Distribution of multidrug resistance (MDR) patterns in clindamycin-resistant *S. agalactiae* (n=178; MDR ≥3 drug classes resistant).

All 178 strains were sensitive to penicillin, ampicillin, linezolid, vancomycin, and tigecycline. Except for clindamycin, the resistance rates to erythromycin, levofloxacin, and tetracycline were 95.5% (170/178), 60.1% (107/178), and 56.7% (101/178), respectively. Multidrug resistance (MDR) analysis showed that 91.6% (163/178) of the isolates were MDR (defined as resistance to ≥3 classes of antimicrobial agents). The predominant MDR patterns were clindamycin (Gaddy et al., 2024) + erythromycin (ERY) + levofloxacin (LEV) (36.5%, 65/178) and CLI + ERY + tetracycline (TET) (31.5%, 56/178), followed by the quadruple-resistance pattern CLI + ERY + TET + LEV (21.9%, 39/178). (**Figure 1**). These findings indicate that erythromycin-clindamycin co-resistance formed the core of the resistance profile, which frequently coexisted with resistance to tetracycline and/or levofloxacin. Collectively, these combinations constituted the primary multidrug resistance phenotypes of the isolates in this study.

### 3.3 Serotypes, sequence types, and CCs distributions

#### 3.3.1 Diversity of molecular epidemiology

In this study, 15 STs were identified through multilocus sequence typing (MLST) analysis, which showed significant polymorphic distribution characteristics. Among them, ST10 (37.6%, 67/178) was the dominant type, whereas ST19 (20.8%, 37/178) and ST529 (16.9%, 30/178) were the second most dominant types. The combined proportion of the three types was 75.3%, which constituted the core epidemic strain in this study. The other minor types, namely ST1, ST862, and ST890, accounted for approximately 3.9% respectively, whereas ST2231 and ST314, which were rare, were each detected in only 0.6% of the samples. Only one strain had an unknown ST.

In terms of the distribution of CCs, our results revealed a clear hierarchical structure. CC12 (38.2%), the dominant clone, corresponded to ST10, whereas CC19 (23.6%) and CC327 (19.1%) were the second most dominant clone groups. ST19 was entirely attributed to CC19, while ST529 mainly concentrated on CC327 (34/36). Additionally, seven strains (approximately 3.9%) could not be classified under a known CC, which may represent a new genetic lineage.

**Figure 2:**
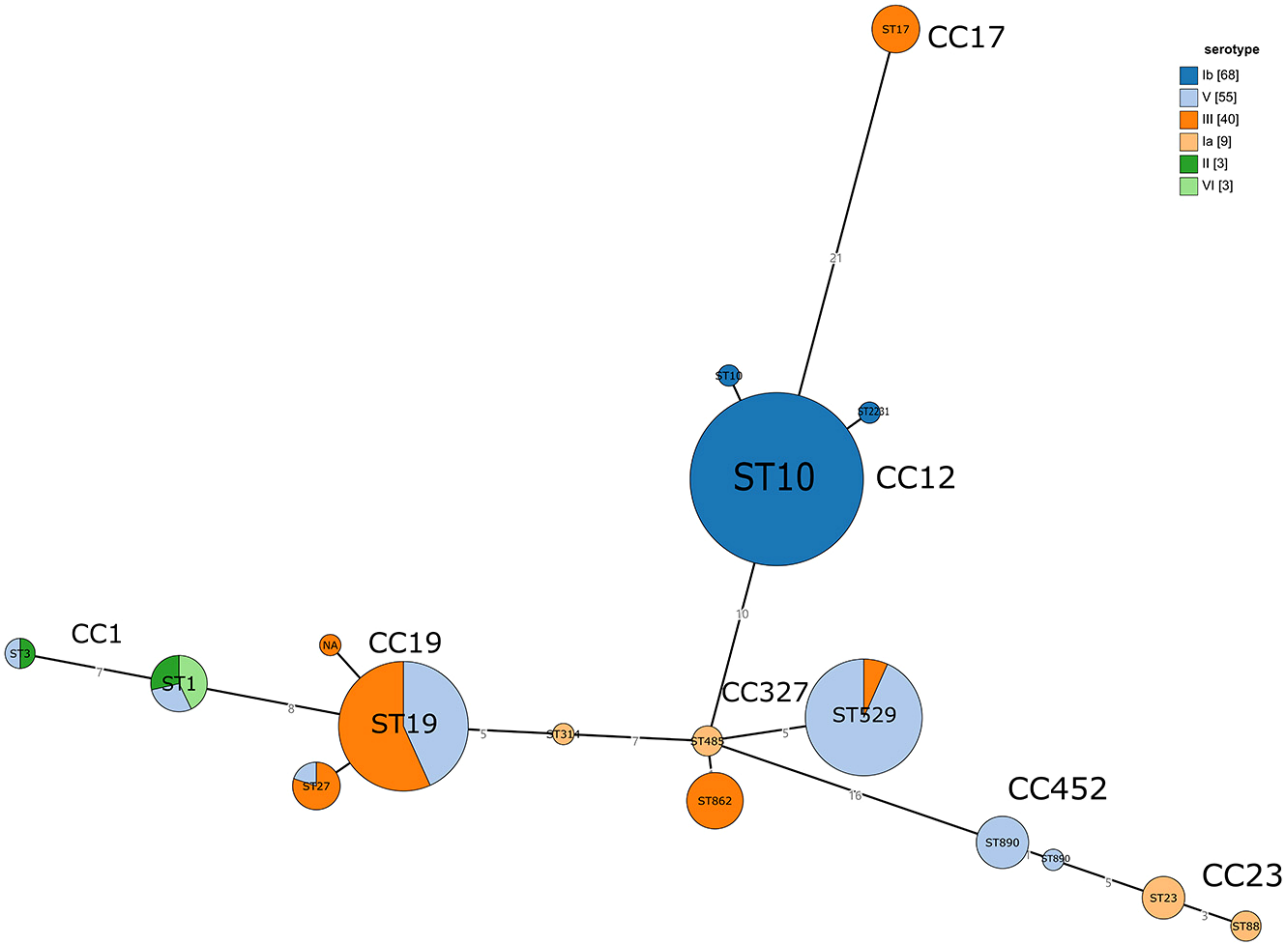
Phylogenetic analysis of the association between population structure (based on MLST) and Serotypes of *S. agalactiae.*.

The distribution of serotypes showed a typical three-level pattern. The dominant serotypes, Ib (38.2%) and V (30.9%), accounted for 69.1% and were significantly associated with CC12 (ST10-Ib) and CC19/CC327 (ST19/ST529-V), respectively. The secondary serotype was III (22.5%), which mainly corresponded to strains CC17 and CC19. Rare serotypes, such as Ia, II, and VI, each accounted for 1.7%. IV serotype was not detected. Further details are presented in **Table 1**. The evolutionary relationship between the population structure and serotype based on MLST is shown in **Figure 2**.

**Table 1:** Serotype, sequence type, and clonal complex distribution of clindamycin-resistant *S. agalactiae* isolate: total and regional comparisons (Jinan vs. Qingdao, Shandong Province).

Typing category	Type	Total (n=178)	Jinan (n=85)	Qingdao (n=93)	Notes
Sequence Type (ST)	ST1	7 (3.9%)	4 (4.7%)	3 (3.2%)	Belongs to CC1
	ST10	67 (37.6%)	37 (43.5%)	30 (32.3%)	Dominant ST in both regions; belongs to CC12
	ST17	5 (2.8%)	3 (3.5%)	2 (2.2%)	Belongs to CC17
	ST19	37 (20.8%)	17 (20.0%)	20 (21.5%)	Belongs to CC19
	ST23	4 (2.2%)	2 (2.4%)	2 (2.2%)	Belongs to CC23
	ST27	5 (2.8%)	2 (2.4%)	3 (3.2%)	–
	ST2231	1 (0.6%)	1 (1.2%)	Not detected	Jinan-specific ST
	ST3	2 (1.1%)	1 (1.2%)	1 (1.1%)	–
	ST314	1 (0.6%)	Not detected	1 (1.1%)	Qingdao-specific ST
	ST485	2 (1.1%)	1 (1.2%)	1 (1.1%)	–
	ST529	30 (16.9%)	10 (11.8%)	20 (21.5%)	Significantly higher in Qingdao; mainly belongs to CC327
	ST862	7 (3.9%)	6 (7.1%)	1 (1.1%)	–
	ST890	7 (3.9%)	Not detected	7 (7.5%)	Qingdao-specific ST
	ST88	2 (1.1%)	Not detected	2 (2.2%)	Qingdao-specific ST
	Unknown	1 (0.6%)	Not detected	1 (1.1%)	–
Clonal Complex	CC1	9 (5.1%)	5 (5.9%)	4 (3.2%)	Mainly corresponds to ST1/serotype Ia
	CC12	68 (38.2%)	38 (44.7%)	30 (32.3%)	Dominant CC in both regions; mainly corresponds to ST10/serotype Ib
	CC17	5 (2.8%)	3 (3.5%)	2 (2.2%)	Mainly corresponds to ST17/serotype III
	CC19	42 (23.6%)	19 (22.4%)	23 (24.7%)	Mainly corresponds to ST19/serotypes III/V
	CC23	4 (2.2%)	2 (2.4%)	2 (2.2%)	Mainly corresponds to ST23/serotype V
	CC327	34 (19.1%)	12 (14.1%)	22 (23.7%)	Higher in Qingdao; mainly corresponds to ST529/serotype V
	CC452	7 (3.9%)	3 (3.5%)	4 (4.3%)	Scattered serotype distribution
	NA	7 (3.9%)	4 (4.7%)	3 (3.2%)	No corresponding CC identified
Serotype	Ia	9 (5.1%)	3 (3.5%)	6 (6.5%)	Mainly belongs to CC1
	Ib	68 (38.2%)	38 (44.7%)	30 (32.3%)	Dominant serotype in Jinan; mainly belongs to CC12
	II	3 (1.7%)	2 (2.4%)	1 (1.1%)	Scattered CC distribution
	III	40 (22.5%)	23 (27.1%)	17 (18.3%)	Mainly belongs to CC19/CC17
	V	55 (30.9%)	18 (21.2%)	37 (39.8%)	Dominant serotype in Qingdao; mainly belongs to CC19/CC327
	VI	3 (1.7%)	1 (1.2%)	2 (2.2%)	Scattered CC distribution

#### 3.3.2 Molecular and epidemiological characteristics of *S. agalactiae* in Jinan and Qingdao

Among the 85 strains isolated from Jinan, ST10 was the most dominant, accounting for 43.5%. ST10, ST19, and ST529 formed a “tripartite confrontation” pattern, accounting for a combined 75.3%. ST2231 was detected only in Jinan, suggesting the possible existence of region-specific variant strains. In Qingdao (n = 93), ST10 (32.3%) formed a triangular equilibrium with ST19 and ST529 (21.5% each), and no single dominant type was observed. In terms of the diversity index, 13 STs were detected in Qingdao and 11 in Jinan. ST890 accounted for 7.5% of the total, which is a regional characteristic. The detection rate of ST88 was 2.2%, and its distribution may be related to the adaptability of specific hosts.

Regarding the distribution of CCs, CC12 was the most dominant in both regions (44.7% in Jinan vs. 32.3% in Qingdao); however, there were differences in the dominant serotypes (Ib in Jinan vs. V in Qingdao). For CC19, the proportions were similar in both regions (23.5% in Jinan vs. 24.7% in Qingdao), with both characteristics of dual serotypes III and V. CC327 was more prominent in Qingdao (23.7% vs. 14.1% in Jinan) and was strongly associated with the V serotype.

Serotype Ib was the most prevalent serotype in Jinan, accounting for 44.7% (38/85) of the cases, followed by serotypes III (27.1%) and V (21.2%). Meanwhile, V accounted for 39.8% (37/93) of the Qingdao isolates and was the dominant serotype. Serotypes Ib (32.3%) and III (18.3%) were then followed up. The detection rate of type Ia was 6.5% in Qingdao, which was 1.86 times that in Jinan, indicating regional specificity. No serotype IV strains were detected in either region. Further details are presented in **Table 1**.

**Figure 3:**
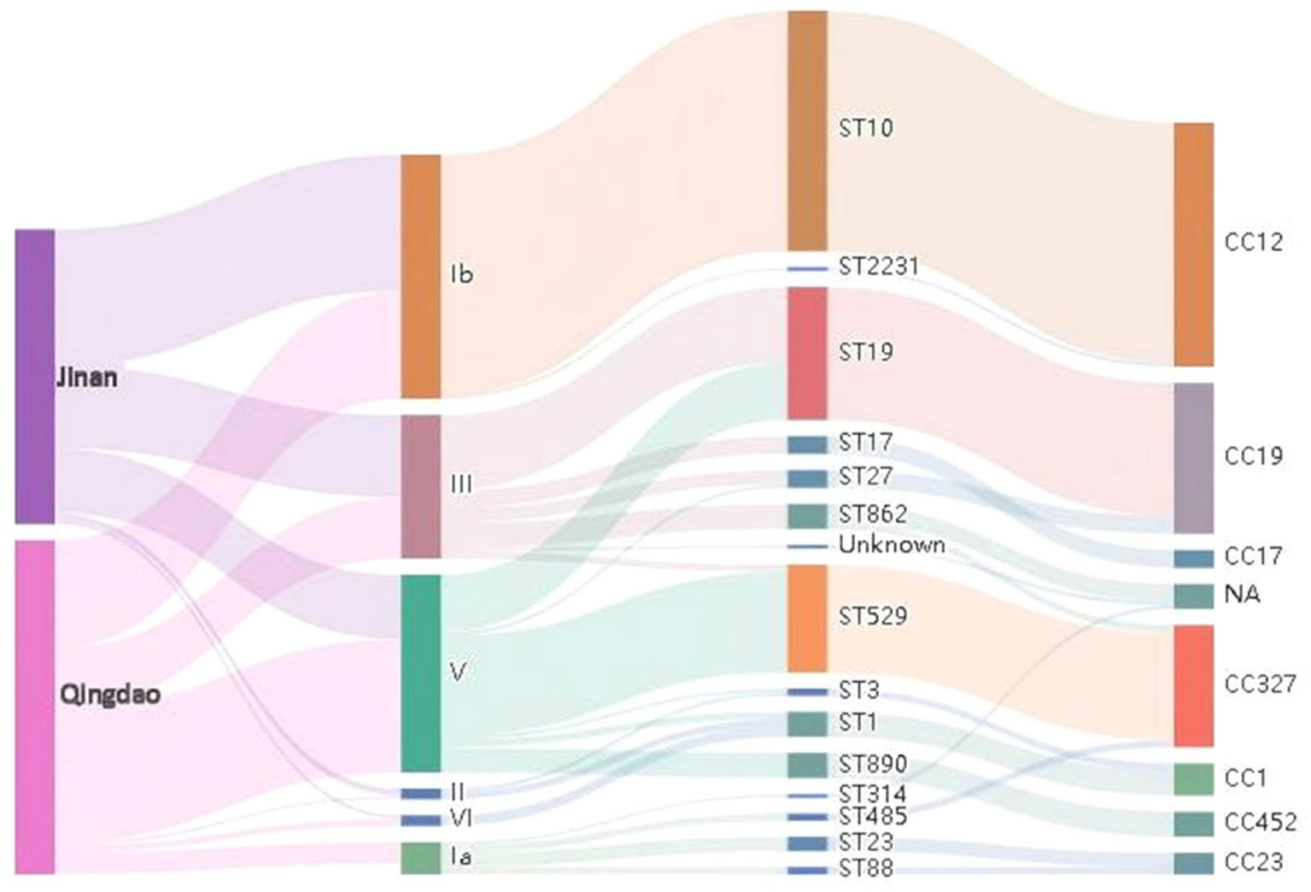
Sankey diagram showing associations among serotypes, MLST, and clonal complexes of clindamycin-resistant *S. agalactiae* strains in Jinan and Qingdao Regions.

In Jinan, the molecular transmission chain showed serotype Ib as the core starting point and was associated with CC12 through the dominant type ST10, forming the dominant transmission chain of “Ib-ST10-CC12.” Serotype III was mainly connected to CC19 via ST19, forming the secondary transmission route of “III-ST19-CC19.” However, the strains from Qingdao presented a transmission pattern centered on serotype V. They were strongly associated with CC327 through ST529 to construct the dominant chain of “V-ST529-CC327.” The proportion of “Ib-ST10-CC12” was relatively reduced compared to that in Jinan. Further details are shown in **Figure 3**.

### 3.4 Analysis of antimicrobial resistance genes

#### 3.4.1 Overall distribution of resistance genes and characteristics of multidrug-resistant strains

Sixteen antibiotic resistance genes were detected, and all strains showed obvious multidrug-resistant characteristics. The structural resistance gene *mreA* was widespread (100%, 178/178), constituting the basis for inherent resistance in *S. agalactiae*. *ermB* was dominant (80.3%, 143/178), whereas the proportions of the efflux pump genes *mefA* and *msrD* were each 17.4% (31/178). *tetM* (31.5%, 56/178) and *tetO* (24.2%, 43/178) were the main determinants of resistance, and the detection rate of *tetS* was extremely low (1.7%, 3/178). Meanwhile, aminoglycoside-modified enzyme genes showed gradient distribution characteristics. Among them, *aph(3’)-III* was the most common (18.5%, 33/178), significantly higher than *ant(6)-Ia* (9.0%, 16/178) and *aac(6’)-aph(2”)* (8.4%, 15/178). The detection rates of *gyrA* and *parC* mutations were both approximately 60.1% (107/178).

Among the 91.6% (163/178) MDR strains, resistance genes exhibited characteristic combination patterns: In strains with the “CLI+ERY+LEV” resistance pattern, the co-occurrence rate of *ermB* and *gyrA/parC* dual mutations reached 92.3% (59/64); in strains with the “CLI+ERY+TET” resistance pattern, the synergistic carriage rate of *ermB* and *tetM/tetO* was 87.5% (49/56); all “four-drug resistant” strains (CLI+ERY+TET+LEV) simultaneously carried *ermB*, *tetM/tetO*, and *gyrA/parC* mutations, among which 94.9% (37/39) were positive for the aminoglycoside resistance gene *aph(3’)-III*, indicating a broader resistance spectrum.

#### 3.4.2 Relationship between drug-resistance genes and CCs

**Figure 4:**
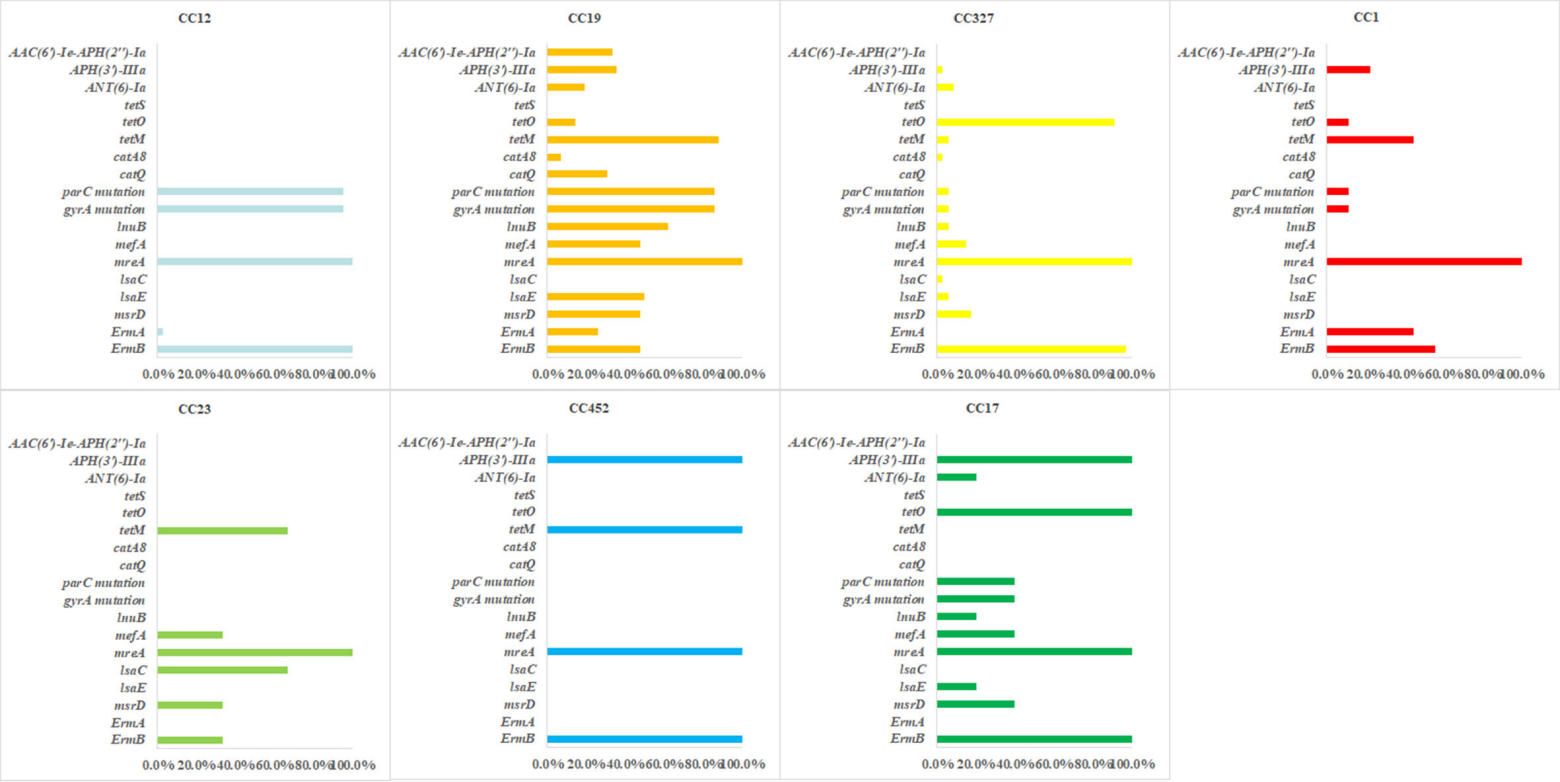
Relationship between antimicrobial resistance genes and clonal complexes of *s. agalactiae* strains.

Significant differences in resistance gene profiles were observed among different clonal complexes. All CCs harbored *mreA*, while other resistance genes showed clone-specific distribution (**Figure 4**).

CC12 was dominated by *ermB* (100%) and *gyrA/parC* mutations (95.6%), exhibiting dual resistance to macrolides and quinolones. The *tetM/tetO* were not detected. CC19 was characterized by the co-occurrence of *tetM* (88.1%) and *parC/gyrA* mutations (both 85.7%), accompanied by a variety of macrolide/lincosamide resistance genes, thus exhibiting significant multidrug resistance. It revealed relatively concentrated resistance gene profile in CC327, with high-frequency co-occurrence of *ermB* (97.1%) and *tetO* (91.2%), while the detection rate of *gyrA/parC* mutations was only 5.9%. CC17 was the single fully resistant clone and all strains carried the *ermB/tetO/aph(3’)-IIIa* combination. CC23 was characterized by *lsaC*-mediated (66.7%) specific lincosamide resistance, with the detection rate of the *ermB* was 33.3%.

#### 3.4.3 Analysis of drug resistance gene evolution based on the minimum spanning tree

**Figure 5:**
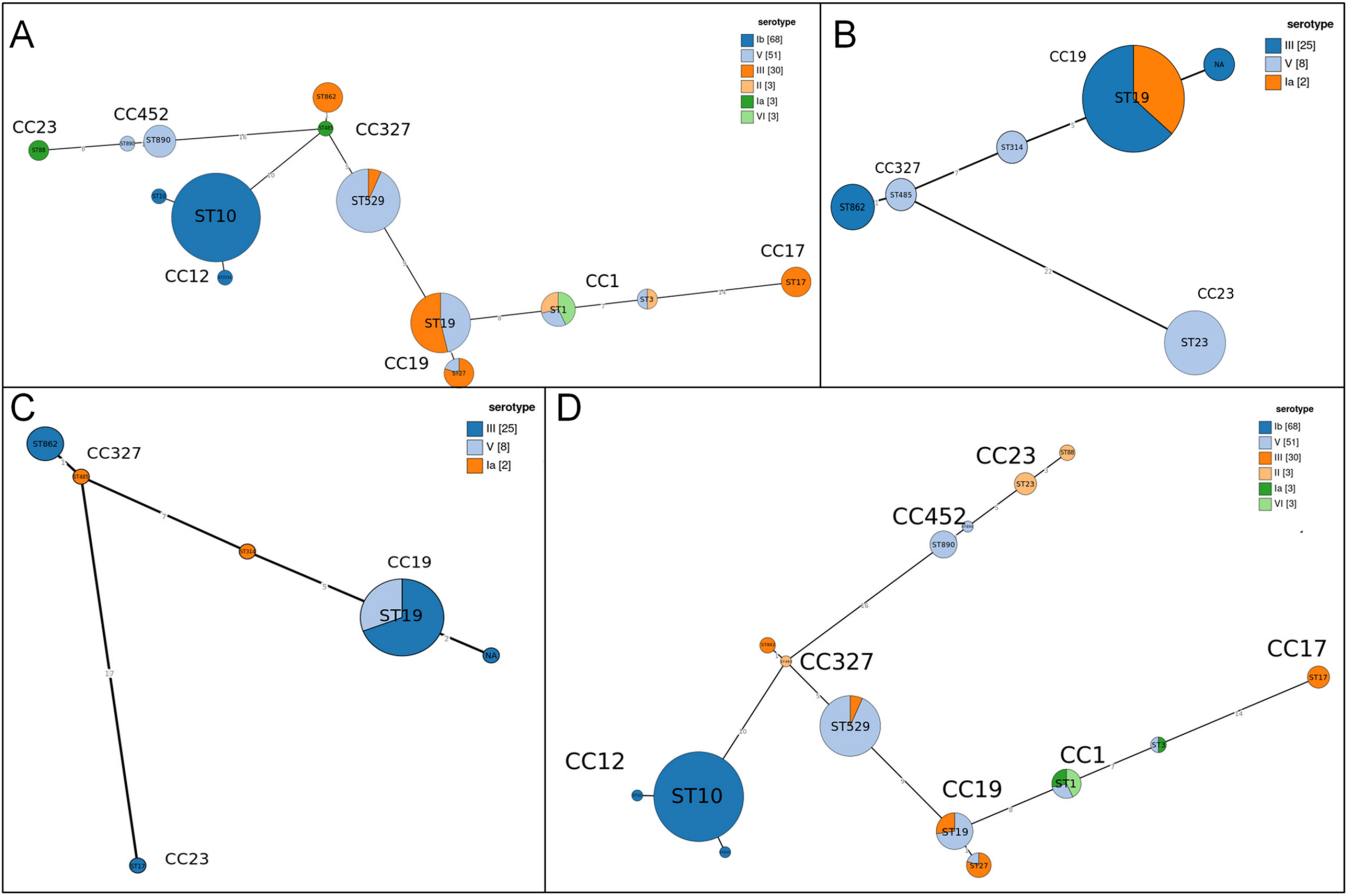
Phylogenetic distribution of *s. agalactiae* strains across clonal complexes, stratified by *erm* and *inu(B)*carriage status. Panels denote strain subsets: **(A)***erm*-positive, **(B)***erm*-negative, **(C)***lnu(B)*-positive, **(D)***lnu(B)*-negative.

Using the minimum spanning tree algorithm, we systematically revealed the evolutionary characteristics of *erm* and *lnuB* in different clone populations (**Figure 5**). The population structure of *erm*-positive strains identified CC12 (ST10, serotype Ib) as the predominant clone, implying a specific advantage in *erm* spread. CC19 (ST19) and CC327 (ST529) displayed serotype diversity (III and V), supporting multiple independent *erm* acquisition events. In contrast, the *erm* exhibited a dispersed distribution in CC1, pointing to dissemination via independent evolutionary events or horizontal gene transfer rather than clonal spread. The *lnuB*-positive strains were concentrated within specific lineages (e.g., CC327, CC19, and ST19) with certain (e.g., CC19) demonstrating serotype diversity (III and V). In contrast, *lnuB*-negative strains were widely dispersed across multiple, diverse clonal complexes. Both *lnuB-*positive and negative strains coexisted in CC19.

### 3.5 Analysis of virulence genes

#### 3.5.1 Overall distribution and functional characteristics of virulence genes

**Figure 6:**
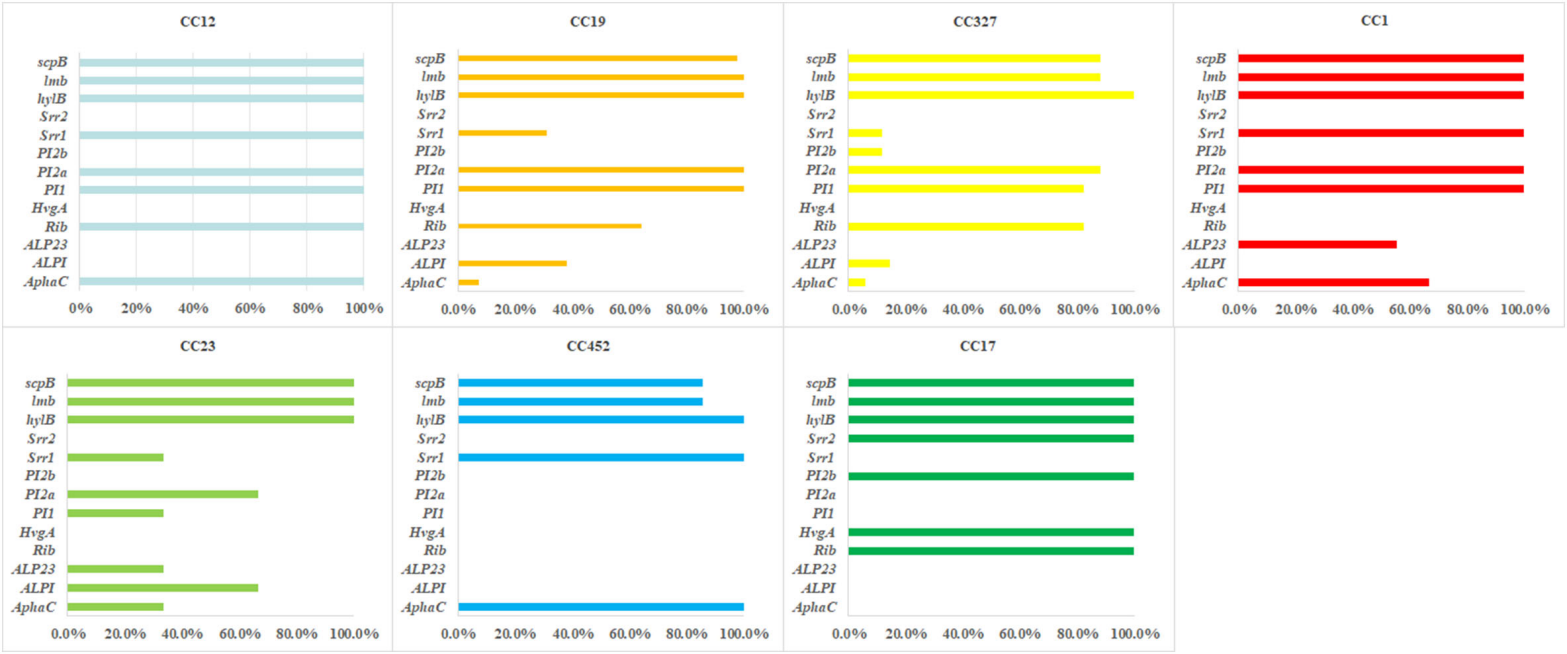
Relationship between virulence genes and clonal complexes of *s. agalactiae* strains.

Virulence gene analysis revealed that the key pathogenic factors were highly conserved. All strains carried *cylE*, *hylB*, and *pavA*. Belonging to the immune escape system, α-protein family genes (98.9%, 176/178) and complement resistance protein gene *scpB* (90.4%, 161/178) were nearly ubiquitous. The fibronectin-binding protein encoded by *fbsA* (99.4%, 177/178) and laminin-binding protein by *lmb* (92.7%, 165/178) formed a “dual-core” adhesion system, which collaborated with the pilus structure protein encoded by *PI* (94.9%, 169/178) and surface protein by *Srr* (63.5%,113/178) to mediate host colonization. The hemolysin gene *hylB* was detected in all the strains. Together with the CAMP factor encoded by *cfb* (98.3%, 175/178), they form a key molecular basis for tissue invasion. Additionally, **Figure 6** shows the maximum likelihood phylogenetic tree of SNPS in the core genome and the status of resistance genes and virulence factors.

#### 3.5.2 Clone-specific correlation between virulence genes and CCs

**Figure 7:**
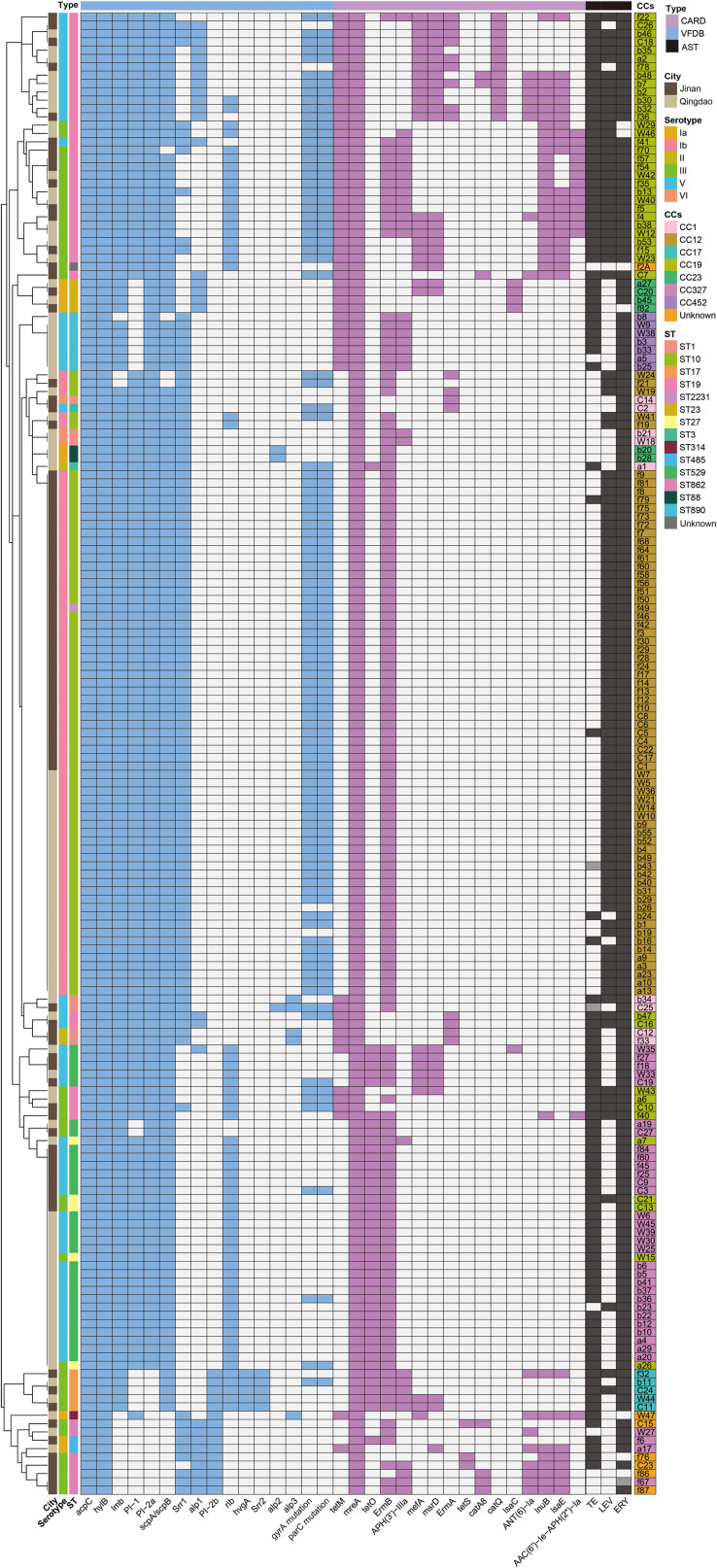
Heatmap of antimicrobial resistance profiles and genotypic characteristics of *s. agalactiae* strains.

Virulence gene profiles of different CCs showed significant clone-specificity, reflecting evolutionary strategies adapted to diverse host microenvironments (**Figure 7**).

CC12 was dominated by alpha protein *AphaC* (100%) and surface protein *Srr1* (100%) and the detection rates of adhesion genes *lmb* and *scpB* both reached 97.1%, while lacking specific virulence factors such as *ALPI* and *ALP23*. The core virulence characteristics of CC327 included the *Rib* (82.4%) and fimbrial genes (*PI1* 82.4%, *PI2a* 88.2%), with balanced expression of adhesion and immune evasion-related genes. CC17 carried signature invasion factors *HvgA* and *PI2b*. The *Srr2* was a CC-specific virulence gene, highly associated with neonatal invasive infections. It was uniquely co-expressed *ALP23* (55.6%) and core virulence factors (*Srr1/lmb/scpB* all 100%) in CC1, with distinct specificity in virulence gene combinations. CC23 highly expressed *ALPI* (66.7%) and *ALP23* (33.3%). Meanwhile, the distribution of other virulence genes was consistent with most CCs. The CC452 showed relatively streamlined virulence profile, with *AphaC/Srr1* detected in 100% of the strains and *lmb/scpB* 85.7%, lacking special signature virulence genes.

#### 3.5.3 Geographical distribution and co-evolutionary characteristics of virulence genes

Dominant clones in Jinan and Qingdao showed geographical specificity in virulence gene distribution. In the dominant clone CC12 of Jinan, the detection rates of *Srr1* and *lmb* (100%) were significantly higher than those of the same CC strains in Qingdao (83.3%). The *Rib* detection rate in the dominant clone CC327 of Qingdao (86.4%) was higher than that in Jinan (66.7%).

Virulence gene expression in multidrug-resistant clones showed co-evolutionary characteristics. In dominant MDR clones such as CC12, CC19, and CC327, the detection rates of adhesion genes (*fbsA, lmb*) and immune evasion genes (*alpha* protein family, *scpB*) were significantly higher than those in non-dominant clones. Among them, the carriage rate of the *PI* fimbrial gene cluster in CC19 MDR strains (95.2%) was higher than that in non-MDR strains (71.4%), suggesting that the resistance and virulence genes might be co-transmitted through mobile genetic elements, enhancing bacterial colonization and pathogenic capabilities.

## 4 Discussion

*S. agalactiae* is a significant pathogen in perinatal infections, with its distribution of serotypes, STs, and CCs showing notable regional and population-specific variations. Over the past decade in China, the predominant serotypes colonizing pregnant women have been III, Ib, and V, with serotype Ia also prevalent in some regions. Serotype III is the most frequently reported in most areas (Cheng et al., 2020; Wang et al., 2023). However, our research indicated that serotype Ib was the predominant one, followed by V and III. These three serotypes collectively accounted for 91.6% of all isolates, highlighting the geographical heterogeneity of serotypes. At the molecular level, ST19 and ST10 are core STs among strains isolated from pregnant women in China, although detection rates of other types vary significantly. Among these, ST19/III is a frequently observed dominant clone combination, while ST10 is often closely associated with serotype Ib (Wang et al., 2023, 2018; Wu et al., 2019). In our region, ST10 was the dominant type, followed by ST19 and ST529. This pattern aligns with findings from Linyi, Shandong Province (Zhou et al., 2024), but differs from most other regions in China where ST19 predominates (Chen et al., 2024; Li et al., 2023; Liu et al., 2022), underscoring inter-provincial variations. Notably, the detection rate of ST17 (often associated with neonatal invasive infections) was relatively low in our study, which is attributable to our focus on routine prenatal screening samples of third-trimester pregnant women. Our results showed that CC12 was the dominant clonal complex, highly corresponding to ST10, with ST10/Ib being the predominant combination. This is largely consistent with a study on *S. agalactiae* colonization and infection in Shanghai (Zhou et al., 2025), but differs from Haikou (Mai et al., 2025). In summary, the distribution characteristics of serotypes, STs, and CCs among *S. agalactiae* strains from pregnant women in this region share common features with the national epidemiological patterns over the past decade, while also exhibiting distinct regional specificity. The data from this study supplement the regional epidemiological profile of *S. agalactiae*, clarify the local epidemiological characteristics in pregnant women, and provide data support for optimizing local *S. agalactiae* screening strategies and vaccine development.

*S. agalactiae* colonization of the genital tract of women during pregnancy is a major risk factor for early-onset neonatal infections, such as sepsis and meningitis (Absalon et al., 2021). Intrapartum antibiotic prophylaxis (IAP) is the standard of care for reducing this risk (Cagno et al., 2012). For pregnant women with penicillin allergy, clindamycin is the recommended alternative agent according to international guidelines (American College of Obstetricians and Gynecologists, 2020). However, the emergence and spread of resistant strains can severely compromise the efficacy of such prophylaxis, posing a significant threat to maternal and infant safety.

Notably, *S. agalactiae* resistance to clindamycin exhibits marked regional heterogeneity globally. The clindamycin resistance rate among reproductive-age women is approximately 40% in South Korea (Shin et al., 2025) and 35.4% in Sri Lanka (Sapugahawatte et al., 2022), while China faces a more severe scenario with a persistently high resistance rate rising by 4%–5% annually (Liu et al., 2023). This regional disparity highlights the urgency of targeted resistance control strategies.

Clindamycin-resistant *S. agalactiae* strains often display multidrug resistance (MDR) phenotypes. A study from Geneva University Hospital reported that among pregnant women, *S. agalactiae* had a 28% clindamycin resistance rate, 30% erythromycin resistance rate, and 92% co-resistance rate to erythromycin and clindamycin (Capanna et al., 2013). A domestic study on clindamycin-resistant *S. agalactiae* showed even higher resistance rates: 96.3% for erythromycin, 62.7% for levofloxacin, and 53.6% for tetracycline, with some strains exhibiting resistance to five antibiotics (clindamycin, erythromycin, levofloxacin, tetracycline, and chloramphenicol) (Liu et al., 2023).

Consistent with these reports, our study of 178 clindamycin-resistant *S. agalactiae* strains found a very high erythromycin resistance rate (95.5%), along with considerable resistance to levofloxacin (60.1%) and tetracycline (56.7%). The prevalent cross-resistance and MDR patterns observed suggest that the regional dissemination of resistance genes under sustained antibiotic selection pressure may have synergistic effects. This underscores the urgent need for targeted surveillance and control strategies to curb the proliferation of these resistant clones.

Ribosomal methylation (MLSB-type resistance) is the predominant mechanism of clindamycin resistance in *S. agalactiae*, mediated by the *erm* gene family (Leclercq, 2002). Among these, *ermB* has the widest global distribution, with a prevalence rate as high as 80%–95% in China. Meanwhile, *ErmA* (5%–15%) and *ermTR* (<5%, predominantly found in Europe) are significantly less common (Liu et al., 2023). This mechanism can be further classified into cMLSB and iMLSB phenotypes. cMLSB results from the continuous expression of the *ermB* gene, accounting for 60%–85% of resistant strains in China, and can be detected directly via routine drug sensitivity tests. iMLSB accounts for 10%–30% of cases in China and requires *erm* gene expression, which is induced by erythromycin and must be confirmed via the D test (Liu et al., 2023). In this study, 89.9% of the strains harbored *erm* family genes (*ermB* or *ermA*), which represent the core mechanism of cross-resistance to clindamycin and erythromycin. As an intrinsic gene of *S. agalactiae* (Clarebout et al., 2001), *mreA* was detected in all strains. The results indicated that the predominant clindamycin-resistant *S. agalactiae* phenotype was cMLSB (64.0%, 114/178), followed by iMLSB (32.6%, 58/178), consistent with previous studies. Drug inactivation and efflux mechanisms (non-MLSB resistance) were primarily observed in CRES phenotype strains exhibiting erythromycin susceptibility and clindamycin resistance. Our study observed that the detection rate of the CRES phenotype was 3.4% (6/178), which is consistent with the globally reported prevalence (2%–8%) (Liu et al., 2023). *lnu(B)* is the primary determinant of the CRES phenotype. *Inu(B)* encodes lincosamide nucleotidyl transferase, which catalyzes the O-adenosylation modification of lincosamide antibiotics, such as clindamycin, inactivating them and mediating clindamycin resistance. As lincosamide nucleotidyl transferase has no effect on macrolide antibiotics such as erythromycin, the strain remains sensitive to erythromycin (Morici et al., 2017). Additionally, *Isa(C/E)* encodes a type II ATP-binding cassette transporter that actively effluxes clindamycin from the cell, thereby reducing the intracellular drug concentration. This mechanism confers resistance exclusively to clindamycin, without affecting erythromycin susceptibility (Si et al., 2015). In our study, among the six CRES phenotypic strains, five simultaneously harbored both *lnu(B)* and *lsaE*, whereas the other strain carried only *lsa(C)*.

Fluoroquinolone resistance in *S. agalactiae* is primarily attributed to mutations in the DNA topoisomerase genes *gyrA* and *parC*. Characteristic mutations in *gyrA* are serine-to-leucine substitutions at position 81 (Ser-81-Leu), whereas those in *parC* involve serine-to-phenylalanine or tyrosine substitutions at position 79 (Ser-79-Phe/Tyr) (Chen et al., 2024). Our study indicated that these two gene mutations were simultaneously detected in all the levofloxacin-resistant strains. This confirms that the combination of mutations at positions 81 in *gyrA* and 79 in *parC* is the key molecular basis for *S. agalactiae* resistance to fluoroquinolone antibiotics.

The mechanism of tetracycline resistance in *S. agalactiae* primarily involves efflux pump-mediated drug efflux and target site protection mediated by ribosomal protection proteins. Proteins encoded by *tetM*, *tetO*, and *tetS* bind to ribosomes, alter their conformation, and block tetracycline binding to the target site, thereby mediating resistance (Blake et al., 2025). Further analysis revealed a differential distribution of these three *tet* genes across distinct CCs. *tetM* and *tetO* co-occurred in CC19, CC327, and CC1, *tetM* alone was present in CC452 and CC23, and *tetO* alone was found in CC17. Nevertheless, CC12 lacked any of the three genes, and *tetS* was absent from all the identified CCs. This clonal specificity in gene distribution suggests that *tet* transmission may be linked to the spread of specific clones, providing molecular epidemiological clues for understanding the evolution and prevalence of tetracycline resistance in *S. agalactiae*.

Although levofloxacin and tetracycline are not routinely used in pregnant women, the high resistance rates of the two antibiotics among clindamycin-resistant *S. agalactiae* strains indirectly reveal that inappropriate antibiotic use in the environment or other populations may exert “cross-selection” pressure on pregnant women. These results provide theoretical and empirical support for antibiotic-tiered management strategies, thereby curbing the evolutionary accumulation and horizontal transmission of resistance genes from their sources.

We revealed the population structure characteristics of *S. agalactiae* by comprehensively analyzing molecular typing, demonstrating both similarities and significant differences with global and domestic epidemiological patterns. Firstly, CC12 (ST10-Ib) accounted for 38.2% of the isolates, holding a dominant position in this study—this differs from reports in South China where CC19 (ST19-III) was predominant (Zhu et al., 2020), but aligns with a Beijing-based study showing high prevalence of ST10-Ib in pregnant women (Chen et al., 2024). Secondly, the prevalence of CC19 (ST19-V) (23.6%) is consistent with Asian regional reports (25%–35%) (Chen et al., 2024). Notably, the ST529-CC327-V combination exhibited a high prevalence (16.9%) in our study, markedly higher than that reported in eastern China (8.3%) (Liu et al., 2023) and central China (5.7%) (Chen et al., 2024), reflecting unique regional selection pressures in Shandong. Geographical variations were also observed in CC17 (ST17-III), which accounted for only 4.5% in our study—significantly lower than in Africa (>60%) (Bob-Manuel et al., 2021) (30) but comparable to other Chinese studies (3%–6%) (Chen et al., 2024; Zhu et al., 2020). The dominant transmission chains differed by region: “Ib-ST10-CC12” (44.7%) in Jinan and “V-ST529-CC327” (39.8%) in Qingdao, with secondary chains (e.g., “III-ST19-CC19” in Jinan) showing varying proportions. This highlights the need for region-specific prevention strategies.

Evolutionarily, the strict correspondence between ST19 and CC19, as well as ST529 and CC327, strongly suggests genetic stability within clonal complexes (CCs) during evolution—likely driven by niche adaptation or restricted horizontal gene transfer (HGT). A novel finding is the Qingdao-specific ST890 (7.5%), which was exclusively associated with serotype V and CC327, forming a “V-ST890-CC327” sublineage not described in prior Chinese or global studies (Chen et al., 2024; Liu et al., 2023; Zhu et al., 2020). This sublineage harbored a unique combination of resistance genes (*ermB* + *tetO* + *aph(3’)-III*) and virulence factors (*Rib* + *PI1*), suggesting recent adaptive evolution in the coastal region. Additionally, rare STs (ST2231, ST314) detected in this study (0.6% each) may represent emerging evolutionary lineages, as they have not been reported in previous Chinese studies (Chen et al., 2024; Liu et al., 2023; Zhu et al., 2020). Furthermore, the coexistence of both *ermB*-positive and -negative strains in CC19, and *lnuB*-positive strains forming a distinct phylogenetic subcluster within CC19/CC327, indicates dynamic resistance gene acquisition/loss—potentially mediated by mobile genetic elements (e.g., transposons, plasmids) (Morici et al., 2017; Si et al., 2015). This clonal-specific resistance evolution pattern, coupled with regional serotype heterogeneity, underscores the complexity of *S. agalactiae* adaptation under antibiotic selection pressure.

The clonal architecture of *S. agalactiae* in our population was strongly associated with distinct antibiotic resistance profiles, revealing evolutionary strategies shaped by selection pressure. The dominant clone CC12 (ST10-Ib) exhibited a streamlined resistance signature—exclusive reliance on *ermB*-mediated macrolide resistance and target-site mutations (*gyrA/parC*) conferring quinolone resistance—without tetracycline resistance determinants (*tetM/tetO*). This suggests a fitness cost or lack of selection for *tet* genes in this successful lineage, which dominates the transmission chain in Jinan. In contrast, CC19 (ST19-V/III) displayed a complex, multidrug-resistant genotype, accumulating diverse resistance genes (*tetM, lnuB, aph(3’)-IIIa, catQ*), which aligns with its role as a major reservoir for horizontal gene transfer in China (Si et al., 2015). Meanwhile, CC327 (ST529-V), the predominant clone in Qingdao, showed a focused resistance profile with high-frequency co-occurrence of *ermB and tetO*, reflecting niche adaptation to coastal regional selection pressures. Notably, the hypervirulent CC17 (ST17-III) clone, though rare (4.5%), consistently carried a ‘full-house’ resistance combination (*ermB/tetO/aph(3’)-IIIa*), reinforcing its concerning profile as a high-risk, broad-resistant lineage associated with invasive neonatal disease (Zhu et al., 2020). These clonally restricted resistance patterns likely reflect sustained antibiotic selection within specific host niches (e.g., CC17 in mother-infant dyads) and underscore the need for clone-aware stewardship.

Regarding virulence genes, our results revealed both highly conserved core virulence factors and clonally specific accessory virulence determinants. The universal presence of core invasion-associated genes (*cfb* [98.3%], *cylE* [100%], *hylB* [100%]) and adhesion genes (*pavA* [100%], *fbsA* [99.4%]) ensures the fundamental pathogenic potential of all strains. This is consistent with the established model of *S. agalactiae* pathogenesis, where a core set of virulence factors is essential for initial host colonization and invasion (Vornhagen et al., 2017). However, significant differences in virulence gene profiles were observed across CCs, reflecting niche adaptation. CC12, the dominant clone in Jinan, was characterized by 100% expression of host cell adhesion factors (*AphaC, Srr1*) and high detection rates of *lmb* (97.1%) and *scpB* (97.1%), which may enhance its mucosal colonization efficiency in the maternal genital tract (Li et al., 2025; Ma et al., 2025). In contrast, the hypervirulent CC17 clone invariably carried the signature neurotropic invasion factors *hvgA* (100%) and the Pilus Island 2b (*PI-2b*) pathogenicity island (100%), a genetic combination strongly and specifically associated with neonatal meningitis and high mortality rates (Almeida et al., 2017; Aznar et al., 2024). These findings confirm a co-evolutionary pattern between clonal lineages and virulence phenotypes, suggesting that successful clones optimize distinct virulence factor combinations to thrive in specific host microenvironments (e.g., CC17 in the neonate, CC12 in pregnant women).

The clonally specific co-distribution of resistance and virulence genes—such as CC12 (*ermB*+g*yrA/parC* mutations + *AphaC/Srr1*), CC19 (*tetM/lnuB* + *PI1*), and CC17 (*ermB/tetO/aph(3’)-IIIa* + *hvgA/PI-2b*)—suggests integrated adaptive strategies under dual selection pressures. The simultaneous enrichment of multidrug resistance genes and key virulence determinants within dominant CCs points to potential “resistance-virulence” synergistic evolution, possibly facilitated by co-localization on mobile genetic elements (e.g., transposons, plasmids) (Shang et al., 2025). This synergy has critical implications: it implies that the most successful, transmissible clones may also be the most resistant and pathogenic. Therefore, surveillance programs must evolve from monitoring resistance alone to integrating molecular typing and virulence gene screening. Targeted monitoring of high-risk “resistance-virulence” clones (e.g., CC12, CC19, CC17) and timely adjustment of empirical prophylaxis guidelines are imperative to mitigate the public health threat posed by these optimized bacterial lineages.

This study revealed the clone-specific evolutionary patterns in the distribution of the *erm* and *lnuB* across *S. agalactiae* clonal clusters. The *erm* was significantly enriched in CC12, CC19, and CC327 (Morici et al., 2017; Wang et al., 2013), a phenomenon driven by both clonal expansion (Wu et al., 2025) and horizontal gene transfer (HGT, observed in the serotype-diverse CC19 and CC327). The frequent association of erm with mobile genetic elements (MGEs) such as transposons and integrative conjugative elements (ICEs) supports its potential for horizontal transfer (Mingoia et al., 2016; Morici et al., 2017; Wang et al., 2013).

Similarly, the distribution of lnuB was highly clonal-restricted, predominantly enriched in CC19 (61.9%). In contrast, lnuB-negative strains were widely dispersed across other clonal clusters. The coexistence of lnuB-positive and negative strains within CC19 indicates microheterogeneity, likely shaped by regional antibiotic selection pressure or independent HGT events (Brenciani et al., 2014; Wu et al., 2025).

Collectively, the distribution of resistance genes in *S. agalactiae* is determined by clonal background, resulting from the interplay of host adaptation, local antibiotic pressure, and MGE-mediated HGT. In clones such as CC19, the co-occurrence of ermB with other resistance genes (e.g., tetM, tetO, aph(3′)-III) underscores their role as multidrug resistance reservoirs (Wang et al., 2013).

Future research should integrate genomic epidemiological approaches to track MGE dynamics and core genome evolution-such as exemplified in mobilome studies, unravel the co-evolutionary mechanisms of resistance and virulence (Shang et al., 2025). Although our research provides insights based on samples from Shandong Province, larger multi-center longitudinal studies across diverse regions of China are needed to fully elucidate the national epidemiological characteristics and evolutionary patterns of resistant *S. agalactiae* clones (Chen et al., 2024; Wang et al., 2023).

In addition, due to the failure to integrate clinical background information of pregnant women and health outcome data of neonates, the clinical association between *S. agalactiae* colonization characteristics and maternal-neonatal health outcomes has not been fully established. Future studies may incorporate multi-dimensional clinical indicators for in-depth analysis, so as to more comprehensively evaluate the clinical significance and potential impacts of *S. agalactiae* colonization. In this study, an unknown ST strain was identified. Subsequent studies will further verify whether the strain represents a novel ST by Sanger sequencing, which constitutes a limitation of this study. If confirmed as a novel ST, this finding will enrich the MLST typing database of *S.agalactiae*. Additionally, investigating its antimicrobial resistance and virulence gene profiles will provide new insights for the epidemiological surveillance of *S.agalactiae* among third-trimester pregnant women in China. Even if it can match the existing ST, that will not affect the overall results and conclusions of this study.

In summary, this study provides two key findings with direct implications for the targeted control of *S. agalactiae*. Firstly, we identified clonal microheterogeneity within high-risk lineages such as CC19, characterized by the co-circulation of *erm/lnuB*-positive and -negative subpopulations. This pattern is consistent with the established role of macrolide/lincosamide selection pressure in enriching resistant subpopulations (Leclercq, 2002; Wu et al., 2025) and the broader principle of antibiotic-driven population differentiation in bacteria (Ågren et al., 2014; Andersson et al., 2019), thereby pinpointing a critical target for focused surveillance. Secondly, we delineated two concurrent dissemination pathways for major resistance genes (*erm* and *lnuB*): clonal expansion of CC12 (the dominant lineage in the Jinan region) and mobile genetic element (MGE)-mediated horizontal gene transfer within CC19 and CC327. This clarifies the dual-track evolution of resistance in local settings and addresses a key gap in understanding regional AMR dynamics.

To translate these insights into practice, a dual-strategy approach is warranted. Dynamic surveillance must evolve beyond tracking dominant clones (CC12, CC19, CC17) to actively monitor resistance gene flux within critical reservoirs like CC19. Concurrently, stewardship to restrict non-essential antibiotic use is vital to reduce the selection pressure fueling both clonal success and gene transfer. Furthermore, intrapartum prophylaxis guidelines should be adapted regionally based on prevailing resistance patterns, including predefined alternatives for clindamycin-resistant strains. Together, these evidence-based, precision measures offer a pragmatic framework to contain the spread of resistance and reduce the risk of mother-to-child *S. agalactiae* transmission.

## Data Availability

The datasets presented in this study can be found in online repositories. The names of the repository/repositories and accession number(s) can be found in the article/**Supplementary Material**.
